# HERVs Endophenotype in Autism Spectrum Disorder: Human Endogenous Retroviruses, Specific Immunoreactivity, and Disease Association in Different Family Members

**DOI:** 10.3390/microorganisms13010009

**Published:** 2024-12-24

**Authors:** Marco Bo, Alessandra Carta, Chiara Cipriani, Vanna Cavassa, Elena Rita Simula, Nguyen Thi Huyen, Giang Thi Hang Phan, Marta Noli, Claudia Matteucci, Stefano Sotgiu, Emanuela Balestrieri, Leonardo Antonio Sechi

**Affiliations:** 1 Department of Biomedical Sciences, Section of Microbiology and Virology, University of Sassari, Viale San Pietro 43b, 07100 Sassari, Italy; m.bo4@studenti.uniss.it (M.B.); simulaelena@gmail.com (E.R.S.); martanoli@outlook.it (M.N.); sechila@uniss.it (L.A.S.); 2 Struttura Complessa Microbiologia e Virologia, Azienda Ospedaliera Universitaria Sassari, 07100 Sassari, Italy; 3 Unit of Child Neuropsychiatry, Department of Medical, Surgical and Experimental Sciences, University of Sassari, 07100 Sassari, Italy; carta.ale84@gmail.com (A.C.);; 4 Department of Experimental Medicine, University of Rome Tor Vergata, 00133 Rome, Italy; chiara.cipriani@uniroma2.it (C.C.); matteucci@med.uniroma2.it (C.M.); 5 Department of Immunology and Pathophysiology, Hue University of Medicine and Pharmacy, Hue City 53000, Vietnam; nthuyen.dhyd24@hueuni.edu.vn (N.T.H.); pthgiang.med@hueuni.edu.vn (G.T.H.P.)

**Keywords:** ASD, human endogenous retrovirus, genes, artificial intelligence

## Abstract

Increasing evidence indicates that human endogenous retroviruses (HERVs) are important to human health and are an underexplored component of many diseases. Certain HERV families show unique expression patterns and immune responses in autism spectrum disorder (ASD) patients compared to healthy controls, suggesting their potential as biomarkers. Despite these interesting findings, the role of HERVs in ASD needs to be further investigated. In this review, we discuss recent advances in genetic research on ASD, with a particular emphasis on the implications of HERVs on neurodevelopment and future genomic initiatives aimed at discovering ASD-related genes through Artificial Intelligence. Given their pro-inflammatory and autoimmune characteristics, the existing literature suggests that HERVs may contribute to the onset or worsening of ASD in individuals with a genetic predisposition. Therefore, we propose that investigating their fundamental properties could not only improve existing therapies but also pave the way for new therapeutic strategies.

## 1. Introduction

Autism spectrum disorder (ASD) is a complex neurodevelopmental disorder characterized by difficulties in social interaction and communication, as well as restricted interests and repetitive behaviors [1]. The exact cause of ASD is still unknown. The data in the literature suggest that environmental factors such as viral and bacterial infections may act as triggers, in addition to a significant genetic component [2,3].

The diagnosis of ASD is currently based on clinical assessment and requires multidisciplinary teamwork. As detailed in the Section 2.1 titled “Characteristics of ASD, Diagnosis, Signs, Symptoms, and Classification”, ASD may manifest as early as six months of age; however, a conclusive diagnosis is typically not made until the child reaches at least 18 months of age. Moreover, the rapid increase in the prevalence of ASD recorded by the Centers for Disease Control and Prevention (CDC) in the last two decades makes the search for early diagnostic biomarkers associated with ASD urgently needed to anticipate therapeutic intervention as early as possible. Many promising biomarkers have been developed for ASD, but, to date, they must be considered preliminary and not validated for their efficacy in the diagnosis and treatment of ASD. These biomarkers considered include prenatal history, genetic background, metabolic, abnormalities in mitochondrial, folate, trans-methylation and trans-sulfuration pathways, immune system, particularly autoantibodies and cytokine dysregulation, autonomic nervous system, and nutritional. Of note, the development of neuroimaging genetics allows the identification of ASD-risk genes that contribute to structural and functional abnormalities in the brain, elucidating mechanisms and pathways that confer genetic risk. Moreover, integrating Artificial Intelligence models with neuroimaging data seems to support an accurate diagnosis, facilitating the identification of early biomarkers for ASD. Despite the efforts to identify such biomarkers and the emerging of promising evidence, at present, the absence of validation studies prevents its use in clinical practice [4,5].

For the majority of affected individuals, the primary causes of psychiatric and behavioral disorders involve multiple common inherited gene variants that, however, individually account for small levels of phenotypic variation. Therefore, many studies have identified endophenotypes, i.e., discrete quantitative clinical traits reflecting genetic likelihood in both patients and their unaffected relatives, to be considered disorder-specific and, perhaps, diagnostically confirmatory [6]. Since they can participate in the concatenation of determinants between genotype and clinical phenotype, endophenotypes are distinct from biomarkers as they can be central for understanding genetic contributions to neurodevelopmental and psychiatric disorders [7].

In psychiatry, endophenotypes can be traits derived from laboratory and clinical measures, such as gene variations and neurocognitive or behavioral deficits, and they are integrated into family genetic and clinical studies. Examples of heritable endophenotypes in ASD can be considered impaired social engagement, as assessed in twin toddlers by ocular gazing while viewing human interactions [8], and hyperserotonemia, although with no unequivocal heterogeneity of results [9]. Very recently, through a 3D novel technique to trace structural brain changes linked to copy number variations (CNVs) at the 16p11.2 region in individuals with ASD, Kundu et al. (2024) identified new endophenotypes, which enabled up to 95% accuracy in predicting 16p11.2 CNV from brain images alone and a dose-dependent brain changes depending on either gene deletion or duplication [10].

In this review, we will discuss the latest findings on the possible impact of human endogenous retroviruses (HERVs) in ASD based on recent discoveries in this field by clinical microbiologists and their potential utility to clinicians, such as biomarkers. Also, we present data that can help us consider ΗERVs not only as a biomarker of ASD but even as a new endophenotype of this neurobehavioral disorder. Besides understanding human neurodiversity, this new endophenotype can suggest that it may be possible to identify children at high risk for autism, thus guiding early strategies to lessen the impact of the deficit and accelerating precision medicine. In fact, we cannot exclude the possibility that these repetitive and transposable elements may serve as therapeutic targets to improve the ASD phenotype, following the model already applied to HERV-K in amyotrophic lateral sclerosis (ALS) [11].

In ALS, reduced expression of the HERV-K subtype HML-2 following antiretroviral treatment has been associated with slower disease progression. HERVs are repetitive elements previously implicated in different types of psychiatric conditions, including schizophrenia, bipolar disorder, major depressive disorder, and attention deficit hyperactivity disorder in ASD [12,13,14,15,16,17,18,19,20].

A comprehensive search strategy was used in the PubMed literature database, covering the last 20 years. The keywords “human endogenous retroviruses”, “HERVs”, and “autism spectrum disorder”, “ASD”, were used to refine the scope of the literature identified in the initial searches.

HERVs are sequences that have been integrated into the human genome as a result of retroviral infections [19]. They make up approximately 8% of the human genome. HERVs contain both coding and non-coding sequences that have different biological functions, ranging from immune response regulation to syncytiotrophoblast formation and gene regulation [20].

In addition to this, it has been observed that the activity of HERVs can be modulated by microorganisms such as *Mycobacteria* and *Herpesviruses* [21,22,23,24,25]; viral and bacterial infections and stress can lead to their dysregulation, potentially contributing to diseases such as those observed in neurodegeneration, pathological inflammation, multiple sclerosis, rheumatoid arthritis, diabetes, celiac disease, and oncogenesis [26,27,28,29,30,31,32,33,34,35,36]. Studying their properties is, therefore, of fundamental importance, as it could improve existing therapies and help to develop new therapeutic strategies for diseases in which HERVs are involved.

In this review, we aim to provide a comprehensive discussion of the HERVs and how they are related to diseases that affect the nervous system. We are going to start by looking at the nature and characteristics of ASD, including its signs, symptoms, and classification. This is followed by a description of the environmental and genetic risk factors involved in the development of autism. There will also be a report on the epidemiology of ASD in Sardinia, an island in the Mediterranean Sea characterized by a high incidence and prevalence of autoimmune diseases compared to other regions of Italy and Europe, such as adult and pediatric multiple sclerosis and chronic inflammatory demyelinating polyneuropathy [37,38,39,40].

We will consider the proposed mechanisms by which HERV activity may be modulated by microorganisms and how its dysregulation contributes to the pathology of autism. In addition, we will discuss the neurobiology of ASD and analyze the role and activity of HERVs in this condition. Finally, we discuss the future direction of research into HERVs and ASD, including the use of Artificial Intelligence and Mendelian randomization [41].

## 2. Autism Spectrum Disorder

### 2.1. Characteristics of ASD, Diagnosis, Signs, Symptoms, and Classification

ASD is a neurodevelopmental disorder characterized by impairment in social communication and restricted and repetitive behavior or interests [42]. The clinical presentation of ASD is highly heterogeneous, which justifies the classification of ASD as a spectrum disorder. The severity of symptoms can vary from mild to severe, with manifestations ranging from intellectual disabilities and language impairments, to, in some cases, average cognitive functioning accompanied by significant difficulties in communication with social purpose [1,43].

The global prevalence of ASD is approximately 1%, with significant variation between countries. In the U.S., the rate has risen to 1 in 36 children [44,45]. Studies show variable rates, with some regions reporting much lower prevalence, largely due to changes in diagnostic criteria, enhanced diagnostic capabilities, and increased awareness [44,45,46]. The estimated prevalence of ASD is higher in males than in females at a ratio of approximately 4:1 [42,45,47]. Recent studies suggest that this difference in prevalence could partly be attributed to the females’ ability to mask their social difficulties. This capacity allows many females to blend in social environments more effectively, which may delay or hinder their diagnosis, contributing to underdiagnosis in females with ASD [48,49,50].

The diagnosis of ASD is clinical and requires a multidisciplinary team of clinicians, involving figures such as psychiatrists, psychologists, and specialists in neurodevelopmental disorders [51]. The diagnosis consists of detailed behavioral observation and anamnesis, focusing on the individual’s developmental history and current behavior [52]. To assist clinicians in making an accurate diagnosis, several diagnostic tools are available. These tools are generally divided into two categories: parental interviews and direct observations of the patient. Parental interviews, such as the Autism Diagnostic Interview-Revised (ADI-R) [53] and the Childhood Autism Rating Scale (CARS) [54] [NO_PRINTED_FORM], provide critical insights into the developmental history.

For direct behavior assessment, the most used tool is the Autism Diagnostic Observation Scale (ADOS-2) [55]. These instruments are designed to assess the core symptoms of ASD, including impairments in social communication and restricted, repetitive behavior [1,52,56,57,58].

Recent advancements in ASD diagnosis also include emerging technologies such as genetic analysis, neuroimaging (fMRI, MRI), and Artificial Intelligence (AI), which hold promise for earlier and more precise diagnoses. These approaches, even if still in the development and research phase, could potentially enhance the accuracy and timing of ASD identification by supplementing traditional behavioral assessment [59].

First signs of ASD can emerge as early as 6 months of age [60]; despite this, a definitive diagnosis cannot be made before 18 months of age [61,62]. These early indicators often include impairments in social communication, such as reduced eye contact when gazing at an adult’s face, limited smiling or vocalizing with eye contact, a lack of initiation of joint attention, and a diminished response to hearing one’s name. Additional deficits in social communication can include delayed language development, a lack of gestures, reduced social reciprocity (e.g., not engaging in typical back-and-forth interaction), difficulties in forming relationships, and abnormalities in understanding the intent of others [63].

Altered social communication is often associated with sensory processing differences, such as hypersensitivity or hyposensitivity to visual, auditory, or tactile stimuli (e.g., ignoring loud noise, showing little interest in new objects, or not responding to pain) [64].

Additionally, restricted and repetitive behavior are hallmark features of ASD. These may include stereotyped motor movements, repetitive use of objects, unusual sensory interests, rigid adherence to routines, verbal rituals, and neologism or idiosyncratic language [65].

In some cases, children with ASD may experience a regression in previously acquired skills, such as speech or social interaction, during their early development [60,66]. Recognizing early signs is fundamental to initiating timely interventions, which can lead to improved outcomes [67].

**Figure 1:**
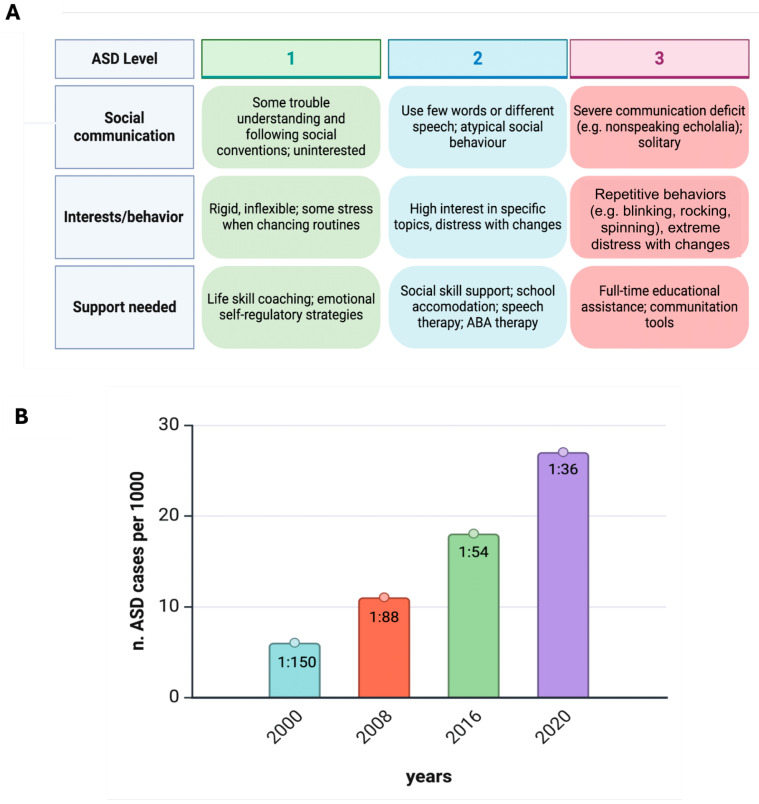
Autism at a glance. (**A**) Diagnosis and treatment of ASD is based on functioning in two domains (DSM-5-TR, 2022). Moreover, a person with ASD can have different levels across the two domains, e.g., Level 1 for social communication and Level 2 for restricted/repetitive behaviors. (**B**) Prevalence estimates of ASD in USA children aged 8 years during the past two decades. From the Autism and Developmental Disabilities Monitoring (ADDM) Network, U.S. Department of Health and Human Services, Centers for Disease Control and Prevention. ASD estimates in 8-year-old children in the USA have increased markedly, from 6.7 per 1000 (one in 150) in 2000 to 27.6 per 1000 in 2020 (one in 36). The sex ratio (boys/girls) is 3.8:1 [44]. Figure created with Biorender.com (https://www.biorender.com/).

The classification in the DSM-5 for ASD (Figure 1) is structured around the individual’s need for support, which is divided into three levels based on the severity of core symptoms in social communication and restricted, repetitive behaviors [42,67]: Level 1: requiring support; Level 2: requiring substantial support; and Level 3: requiring very substantial support [68]. In addition, the DSM-5 includes two critical specifiers that are necessary to refine the diagnosis of ASD:

1. The presence or absence of intellectual impairment;
2. The presence or absence of language impairment.

These specifiers help to further categorize individuals based on their cognitive and linguistic abilities, which can vary greatly among people with ASD. For this reason, the diagnostic protocol must include a comprehensive evaluation of both intellectual and language functioning. This allows for a more personalized and accurate diagnosis, ensuring that clinicians consider the full spectrum of abilities and challenges beyond the core symptoms of ASD [69,70].

These levels are designed to guide clinicians in understanding the amount of support an individual may need in his daily life [47].

However, it is important to notice that this classification system primarily focuses on core ASD symptoms and does not explicitly account for common comorbidities such as attention-deficit/hyperactivity disorder (ADHD) [71], intellectual disability, or mood disorders. These comorbidities often significantly impact the individual functioning and support needs but are considered separately in the diagnostic process [72].

Including these evaluations is particularly important for designing appropriate intervention plans for the best outcomes and treatment options that can include chronic behavioral, psychosocial, pharmacological, and complementary therapies, which cater to the individual’s specific symptomatology [73].

### 2.2. Epidemiology of ASD in Sardinia

Epidemiological studies on ASD in Sardinia are limited but reflect similar trends to global increases in ASD prevalence. The current estimates, based on some large-scale study groups [74], suggest that ASD affects approximately 1–2% of the population, which aligns with global data. Factors influencing the prevalence in Sardinia, as in other regions, include genetic susceptibility, environmental factors, and possibly underdiagnosis in previous years. Sardinia has a unique genetic landscape due to its geographic isolation, which has prompted researchers to investigate potential genetic links to neurodevelopmental disorders like ASD within the population. However, no specific, large-scale epidemiological studies solely focused on Sardinia have been highlighted in the recent literature.

The role of genetic and immune system factors is of particular interest in Sardinia. Recent research has explored maternal immune activation and other genetic predispositions as contributors to ASD in the region, reflecting broader global research trends that point to both genetic and environmental causes of ASD [75].

### 2.3. Etiopathogenesis of ASD: The Role of Environmental and Genetic Factors

The precise mechanisms underlying the combined influence of various etiological factors in ASD remain inadequately understood. Numerous factors have been associated with the development of ASD, including environmental pollution, the accumulation of metal ions, exposure to pesticides, viral and bacterial infections, maternal age, immune deficiencies, maternal–fetal interactions, alterations in gut microbiota, and changes in gene expression and mutations.

It is hypothesized that heavy metals may play a role in the pathogenesis of ASD through epigenetic mechanisms. Specifically, exposure to heavy metals during early development in children may have significant epigenetic effects by influencing DNA methylation, leading to the dysregulation of DNA methyltransferase activity [76].

This dysregulation can alter gene expression, potentially contributing to the risk of ASD development. Additionally, exposure to airborne pollutants containing heavy metals may trigger oxidative stress and inflammatory responses. Heavy metals disrupt enzymatic function and cellular signaling processes, resulting in the production of reactive oxygen species (ROS) and mediating autoimmune responses, thus amplifying inflammatory pathways and cellular damage [77,78]. Finally, scientific studies have demonstrated that heavy metals may accumulate in the central nervous system of individuals with ASD, likely due to a reduced ability to expel these toxic substances. This accumulation can lead to neurotoxicity, contributing to the neuronal dysfunction observed in ASD patients [79].

In addition, there is growing evidence that imbalances in the gut microbiota have a profound effect on many diseases, and recent findings have observed the same trend in ASD. In fact, the gut microbiota performs a wide range of functions, from regulating the immune system to controlling protein metabolism and the breakdown of short-chain fatty acids. It also plays a role in the production of essential vitamins and metabolites. Additionally, it is crucial for detoxifying harmful substances and digesting indigestible dietary fibers. A healthy gut microbiota is vital in preventing the colonization of pathogenic microorganisms, acting as a key factor in maintaining the balance between health and disease [80,81].

It has been observed that in addition to cognitive and behavioral problems, patients with ASD may experience gastrointestinal problems such as abdominal pain and bloating, digestive problems, constipation, diarrhea, reflux, inflammatory conditions, food intolerance, or signs of irritable bowel syndrome (IBS) [82]. This condition can complicate clinical management and contribute to behavioral problems. Changes in the composition of the gut microbiome have also been shown to correlate with the severity of the condition. Over the past decade, it has been recognized that the gut microbiome plays a key role in regulating the gut–brain axis, influencing neuroimmune pathways, and interacting directly with the brain. This suggests that it may play a role in the onset and progression of ASD [83,84,85,86,87]. In addition, the gut microbiota also affects plasma levels of 5-hydroxytryptamine (5-HT). Studies have shown that germ-free (GF) mice have reduced serum 5-HT levels, but colonization of these mice restores serum and colonic 5-HT levels [88].

It has been shown that the composition of the gut microbiota is influenced by genetic and environmental factors in a continuous process that can begin in the womb and continue throughout a person’s life. There is increasing evidence that changes in the gut microbiota during the prenatal and postnatal periods have an impact on nervous system function and behavior. The effects could be at the molecular level; epigenetic mechanisms include DNA methylation, histone modification, and snRNA, which regulate which genes are activated and which are silenced. In addition, unfavorable conditions due to stress can trigger a response that has an effect on the hypothalamic–pituitary–adrenal (HPA) axis. Early stimulation of the immune system by bacterial or viral infections can also reprogram brain circuits in an undesirable way, leading to the development of signs associated with ASD [89]. Thus, altering the composition of the gut microbiota can lead to increased inflammation and intestinal permeability. Several mechanisms have been implicated in the bidirectional communication between the gut microbiota and the brain, including vagus nerve signaling, immune activation, tryptophan metabolism, and the production of microbial metabolites and neurometabolites. For example, paracresol (p-cresol) and the derivative p-cresyl sulphate were found to be elevated in the sample of children with autism. Also, certain bacteria, such as *Clostridium difficile* and specific *Lactobacillus* species, can ferment tyrosine and produce p-cresol via the enzyme p-hydroxyphenyl acetate decarboxylase. It is suggested that elevated levels of p-cresol, along with factors such as intestinal disorders, antibiotic use, and abnormal gut permeability, which are conditions that can lead to excessive p-cresol production, may contribute to the worsening of autism symptoms and related dysfunctions in people with ASD [90,91].

Other than that, other types of bacteria, such as *Sutterella*, appear to play an important role in ASD [92]. Research has found significantly higher levels of *Sutterella* in the gastrointestinal biopsies of children with ASD who have digestive problems compared to those without gastrointestinal problems. This suggests that *Sutterella* may be a triggering factor in ASD, given its higher presence in the gut microbiota of many children with autism and related digestive disorders. Additionally, higher levels of *Sutterella*, as well as *Desulfovibrio* and *Ruminococcus* torques, have been detected in the feces of children with ASD compared to control groups. In contrast, *Prevotella*, a species linked to colon health, was found in lower abundance in the gut microbiota of children with ASD. These findings suggest that the development of ASD might be associated with an imbalance in gut microbiota, where an increase in *Sutterella* could occur in conditions where it is typically less prevalent [93,94,95].

However, the exact cause of changes in the microbiota is also unknown, as children with ASD often display altered routines and eating habits, which can affect the microbiota. For this reason, several nutritional strategies, including gluten- and casein-free diets, the use of probiotics and prebiotics, as well as fecal microbiota transplantation (FMT), have been suggested to help reduce gastrointestinal problems in individuals with ASD [96,97,98].

Recent scientific studies have investigated potential risk factors for ASD, focusing on environmental factors, as well as maternal infections during pregnancy or the use of medications, such as valproic acid.

Certain viral infections during pregnancy, contracted by the mother during pregnancy, such as *rubella*, *herpesvirus*, and *cytomegalovirus*, but also other bacterial infections that cross the blood–brain barrier, have historically been associated with an increased risk of congenital abnormalities and developmental disorders in the child, as confirmed in a recent metanalysis [99]. Interestingly, a recent Swedish study on more than 500,000 children suggested that pregnancy infections, severe enough to require specialist care and with the exclusion of COVID-19, increase the risk of intellectual disability in the child but perhaps not significantly that of ASD [100]. ASD outcome can strongly depend on susceptibility factors to a pro-inflammatory milieu of the mothers [75,100].

As for the use of medication during pregnancy, a direct association between valproic acid, an antiepileptic drug, and ASD condition is known. Prenatal exposure to this medication has been linked to an increased risk of congenital malformations and neurobehavioral disorders, including ASD [101]. Updated guidelines emphasize the importance of avoiding valproic acid use in women of childbearing age unless strictly necessary and when no effective therapeutic alternatives are available.

In sum, it is crucial for pregnant women or for those who are planning a pregnancy to consult their doctor to assess the risks associated with certain medications and to take preventive measures against infections depending on their predisposition to pro-inflammatory milieu.

Genetic risk factors play a crucial role in the development of ASD. Several genetic variants have been associated with ASD, highlighting its complex and multifactorial nature. Here, we reported some of the most studied key genetic risk factors. De novo mutations are genetic mutations that appear for the first time in an individual and are not inherited from parents. Studies have identified de novo mutations in genes that are crucial for brain development, such as CHD8, SCN2A, and SHANK3 [102]. Copy Number Variations (CNVs) involve large segments of DNA being duplicated or deleted, leading to an imbalance in gene dosage. Certain CNVs, particularly deletions on chromosome 16p11.2 and 22q13.3, are associated with an increased risk of ASD [103].

About heritability, family and twin studies have demonstrated that ASD has a strong heritable component, with estimates of heritability ranging from 50% to 90%. Siblings of children with ASD are at a higher risk, and identical twins show a much higher concordance rate for ASD compared to fraternal twins [104].

Gene–environment interaction studies are growing in their strength. In fact, while genetic factors play a significant role, interactions between genes and environmental factors, such as maternal infections, advanced parental age, or prenatal exposure to certain drugs, can further increase the risk of ASD [105].

In this context, genetic and immune system factors are gaining particular interest in the study of ASD in Sardinia. Recent research has focused on maternal immune activation (MIA) and genetic predispositions as key contributors to ASD [75]. MIA, which occurs when a mother’s immune system is activated during pregnancy, can affect fetal brain development, potentially leading to ASD. It has been observed that infection-induced maternal serum cytokines, such as interleukin (IL)-6 and IL-17, can cross the placenta and stimulate the downstream production of other detrimental immune mediators of brain damage in the human fetal compartment, thereby inducing behavioral and cognitive features that closely resemble human ASD features [75]. This mechanism, along with Sardinia’s unique genetic landscape due to its geographic isolation, suggests that both genetic and environmental factors are at play in the region. The findings reflect global trends that underscore the interplay between genetics and environmental exposures, such as infections or immune challenges during pregnancy, in the development of ASD.

Polygenic risk studies showed that ASD is thought to involve multiple genetic variants, each contributing a small amount to the overall risk. Genome-wide association studies (GWAS) have identified hundreds of common genetic variants that are slightly associated with ASD, contributing to the polygenic risk [106].

In some cases, ASD occurs as part of a broader syndrome caused by specific genetic conditions, such as Fragile X syndrome, Rett syndrome, or tuberous sclerosis complex. These syndromes highlight specific genes, like FMR1, MECP2, and TSC1/2, that are directly involved in ASD when mutated [107].

Overall, genetic factors have a central role in the susceptibility to ASD and in its clinical complexity [108,109], as shown by the significant concordance among monozygotic twins compared with their dizygotic counterparts. It is estimated, however, that inherited genes may account for only 20% of ASD cases, as they are also present in neurotypical individuals [110]. Most importantly, the concordance in dizygotic twins is progressively increasing, and several de novo gene mutations are emerging as new susceptibility factors, which imply other influences are operating during gestation [111]. ASD-associated or putatively causative immune genes are extraordinarily abundant in the literature.

An early dispute was about the involvement of the human leukocyte antigen (HLA) in ASD. Class I and class II alleles sharing the third hypervariable region have been associated with ASD in various studies among Caucasians. In our early studies on ASD families (with at least one affected child), we found significant associations with HLA regions mapping classical HLA-A and HLA-B and non-classical HLA-G alleles [112,113].

Later, we studied immune genes and cells, which stand at the maternal–fetal interface that allows the fetus to safely develop in the uterus. Decidual CD56 + CD3- natural killer (NK) cells are the most frequent immune cells at this level during early pregnancy and have immunosurveillance roles, among others [114]. Through interaction between their killer cell immunoglobulin-like receptors (KIRs) and the cognate HLA ligands, NK cells play a crucial role in the fetal protection of the virally infected cells placental cells through either pro-inflammatory or tolerogenic KIR/HLA interaction [115]. We found that pro-inflammatory KIR/HLA are more represented, whereas tolerogenic KIR/HLA gene complexes are less represented in children with ASD and in their mothers [116], suggesting a dysregulated interaction between maternal KIRs and filial HLA-G able to induce a detrimental pro-inflammatory status influencing fetal brain growth [75]. We also found that the tolerogenic HLA-G*01:01 allele is less common, whereas the NK-activating HLAG* 01:05N allele is more frequent in affected children and their mothers than controls [117] and correlated with the extent of behavioral disorders in children with autism [118]. Analogous HLA-G allele distribution is present in a group of women with recurrent miscarriages, which confirms that a pro-inflammatory intrauterine environment may cause miscarriage and may be detrimental to typical neurodevelopment.

Vitamin (Vit.) D is known to have immunomodulatory activity along with neuroprotective functions, and it is particularly important during pregnancy; Vit. D serum reduction in pregnant women contributes to neurodevelopmental disorders and behavioral abnormalities in the offspring. Also, Vit. D serum level in children with ASD is inversely correlated with their language and behavioral scores [119]. Bioactivity of Vit. D largely depends on the activity level of its receptor (VDR), whose gene contains several polymorphisms. In a large cohort of families with ASD children, we found that the Vit. D/VDR complex with low biological activity polymorphism is prevalent in children with ASD and their mothers [120]. Later, we studied the role of the isoforms of the vitamin D binding protein (VBP) genes and found that the GC1f isoform of VBP is associated with reduced Vit. D levels in ASD and is a marker of ASD clinical severity. In fact, the GC1f isoform carriers had higher scores on the Childhood Autism Rating Scale (CARS) and lower scores on the Children’s Global Assessment Scale (CGAS) functioning scales [121]. This genetic approach may be useful to establish the magnitude of therapeutic protocols of Vit. D supplementation.

In conclusion, the genetic architecture of ASD is complex and influenced by environmental exposures, like gestational maternal infections. Recent studies determine the genetic contribution to this environmental effect in a large dataset of ASD patients with or without a history of gestational infections. The genetic correlation indicated a genome-wide difference between the two ASD phenotypes. However, even ASD cases with a history of maternal pregnancy-related infections may be genetically determined, which highlights the relevance of gestational infections to the genetics of ASD [122]. Thus, ASD can be considered a multifactorial disorder.

**Figure 2:**
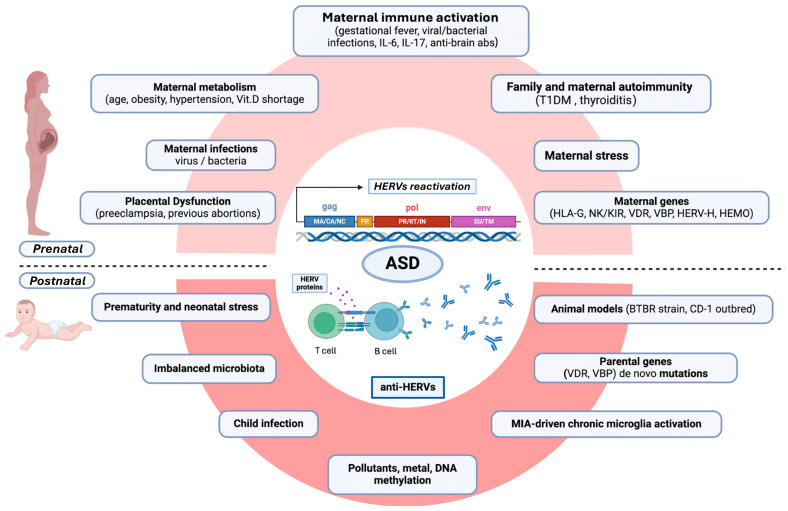
Diagram of the proposed multistep contribution of HERV overexpression and anti-HERV immune responses predisposing to the development of ASD or its clinical severity. Prenatal (upper diagram) and postnatal (lower diagram) roads to HERV-mediated ASD development are traced. Within the prenatal roads, intrauterine inflammatory events or pathogens and other stressful events can trigger a detrimental immune activation at the maternal–fetal interface (maternal immune activation, MIA), favored by a maternal immunogenetic predisposition to MIA (KIR/HLA), familial and personal autoimmunity, and epigenetic forces, such as age and gestational metabolic syndrome. The postnatal steps can take place during early neonatal stress, prematurity, chronic microglial activation, and new infectious events. TDM1 = type 1 diabetes mellitus; HLA-G = human leukocyte antigen G; NK= natural killer cells; KIR = killer cell immunoglobulin-like receptors; VDR= vitamin D receptor; VBP= vitamin D binding protein; HERV-H = human endogenous retrovirus-family H; HEMO = an envelope gene, named human endogenous MER34 (medium-reiteration-frequency-family-34) ORF; IL = interleukin; abs= antibodies. Figure created with Biorender.com (https://www.biorender.com/).

Figure 2 summarizes the content of the text.

### 2.4. Social and Pharmacology Treatments and Interventions

The treatment of ASD requires a multidisciplinary approach, combining social, behavioral, and pharmacological interventions. Social interventions, particularly behavioral therapies, such as Applied Behavior Analysis (ABA), are among the most widely researched and applied approaches. ABA focuses on improving communication and social skills and reducing maladaptive behaviors through reinforcement-based strategies [1]. Early Intensive Behavioral Intervention (EIBI) is a specialized form of ABA for younger children, showing strong evidence for enhancing cognitive functioning and adaptive behavior [123].

The international Evidence-Based Guidelines for the non-pharmacological treatment of Autism recommend various interventions beyond ABA and EIBI.

One highly recommended approach is Naturalistic Developmental Behavioral Interventions (NDBI), which combines behavioral teaching strategies with natural developmental methods to promote social interaction and communication skills in everyday contexts [124].

Another evidence-based technique is the Treatment and Education of Autistic and Communication Handicapped Children (TEACCH), which uses structured visual strategies to enhance independence and environmental understanding in individuals with autism [125]. This approach focuses on structured education that helps organize time and space, reducing anxiety related to predictability.

The Social Communication, Emotional Regulation, and Transactional Support (SCERTS) model is another recommended intervention that emphasizes the development of emotional regulation and functional communication skills while providing environmental support to facilitate social interaction [126]. Lastly, Parent-Mediated Interventions are widely promoted in the guidelines. These interventions involve parents as co-therapists and aim to improve children’s social interaction skills through daily activities guided by specialists [127]. Evidence suggests that active parental involvement can significantly enhance the effectiveness of treatment [128,129].

Pharmacological treatments are often used to manage co-occurring conditions associated with ASD, such as irritability, anxiety, and hyperactivity, but they do not target the core symptoms of the disorder.

Risperidone and aripiprazole, both atypical antipsychotics, have been FDA-approved for the treatment of irritability and aggression in children with ASD [130]. Studies indicate that these medications can reduce behavioral problems but may carry side effects such as weight gain and sedation [43].

Selective Serotonin Reuptake Inhibitors (SSRIs) have been studied for their potential in treating repetitive behaviors and anxiety in ASD, but the results are mixed. While some evidence suggests a reduction in anxiety and obsessive-compulsive behaviors [131], other studies highlight limited efficacy and potential side effects like increased agitation [132].

Methylphenidate, a stimulant medication widely used for attention deficit hyperactivity disorder (ADHD), has shown some efficacy in treating symptoms of inattention, hyperactivity, and impulsivity in children with ASD who have comorbid ADHD. However, the response rate in children with ASD is typically lower, and the risk of side effects, such as irritability, increased anxiety, emotional lability, and worsening of stereotypic behaviors, is higher compared to children with ADHD alone [133].

Guidelines recommend starting at a low dose and monitoring closely for side effects. Although methylphenidate can be effective in managing ADHD symptoms in this population, it should be part of a broader treatment plan that includes behavioral interventions, as it does not address the core symptoms of ASD. Alternatives like atomoxetine may be considered if methylphenidate is not well-tolerated [134].

Recent advances also point toward a growing interest in personalized medicine, where interventions are tailored based on the individual’s genetic, neurobiological, and behavioral profiles [135]. This approach holds promise for the future of ASD treatment, potentially allowing for more effective and individualized therapeutic strategies [136]. In conclusion, the integration of psychological, behavioral, and social, as well as pharmacological treatments, remains critical in managing ASD. Ongoing research continues to explore the best strategies for improving outcomes, with current treatments focusing on addressing co-occurring symptoms while enhancing social functioning and quality of life [137].

## 3. HERVs in Autism Spectrum Disorder

### 3.1. Expression Profile of HERVs and Inflammatory Mediators in Autism Spectrum Disorders

Recent studies have investigated HERVs as possible contributing factors in autism, linking environmental stimuli, epigenetic remodeling, and biological processes in the etiopathogenesis of these disorders. HERV expression has been investigated in peripheral blood mononuclear cells (PBMCs) of drug-naϊve ASD children of two distinct cohorts, including 58 ASD children vs. 58 age- and sex-matched control children. We demonstrated that HERV-H was statistically significantly higher p in ASD children when compared to control children. The highest levels of HERV-H expression were found in younger ASD children, supporting the hypothesis that HERV-H overexpression might be regarded as a potential early biomarker of ASD. This view was even more supported by the fact that ASD children with more severe impairments in Communication and Motor Psychoeducational Profile-3 showed the highest expression levels of HERV-H [138]. In order to investigate the parent-of-origin effects in ASD in terms of HERVs and immune deregulation, a cohort, including 31 ASD families and 14 healthy control families, has been analyzed. ASD children and their mothers share higher expression levels of HERV-H and HEMO (an envelope gene, named human endogenous MER34 (medium-reiteration-frequency-family-34) ORF) [139] and cytokines as tumor necrosis factor alpha (TNF-α), interferon gamma (IFN-γ), and IL-10 compared to related controls. Therefore, the abnormal expression of HERVs and cytokines was not an exclusive trait of autistic children but also of their mothers, suggesting a close mother–child association within ASD families [140].

Finally, the expression of epigenetic effectors known to regulate HERV expression and brain functions has been evaluated in ASD children, demonstrating a correlation between the overexpression of these elements and several HERVs [141].

Results obtained in humans are corroborated by investigation in ASD mouse models, specifically in two preclinical models, the inbred BTBR T + tf/J and valproate-treated CD1 mice [142,143]. BTBR strain is one of the most validated mouse models of autism that recapitulates the core symptoms of the human condition, including disturbances in social interaction, high levels of stereotypy, and an unusual repertoire of ultrasonic vocalizations [144,145,146,147], as well as neuroanatomical features with ASD individuals [148]. CD-1 outbred mice, treated in utero with VPA, show behavioral ASD-like alterations, including early motor hyperactivity, social deficits, and cognitive impairments [149]. Of note, the use of VPA in humans as antiepileptic medication during pregnancy is associated with a significantly increased risk of somatic anomalies, ASD, and other developmental disabilities in the offspring [101]. In both ASD mouse models, higher expression levels of ERVs from intrauterine life up to adulthood in blood and brain compartments have been found in mice with ASD-like phenotype compared to relative controls [142]. Moreover, the aberrant expression of ERVs found in mice with autistic-like phenotype positively correlated with expression levels of proinflammatory cytokines and TLR-3 and TLR-4 in embryos and brain tissues, supporting the interplay between ERVs and immune response [142]. Interestingly, in the valproate-treated mice, the increased expression of ERVs and behavioral alterations were inherited across generations via maternal lineage [143], further confirming the pivotal role of the maternal molecular profile in the acquisition of autistic phenotype. Recently, in a sister strain of BTBR characterized by more prominent core symptoms of autism, an altered epigenetic silencing mechanism was found to lead to increased ERV activity, which increases CNV formation and genome instability. Furthermore, active ERVs, similar to a viral infection, have been shown to evade the integrated stress response and alter the transcriptional mechanism during embryonic development. Overall, the evidence suggests a dual role of ERVs in ASD pathogenesis, driving host genome evolution and altering cellular gene expression, potentially affecting embryonic development [150].

The parallel ERV activation in the two different districts, blood and brain, was also found in the ASD mouse model of MIA induced by prenatal exposure to polyinosinic:polycytidylic acid (Poly I:C) [151], a synthetic double-stranded RNA molecule targeting TLR-3 that mimics viral maternal infection during pregnancy [152]. As such, the altered ERV transcriptional profiles were demonstrated in the placenta and fetal brain tissues, as well as in adult offspring, stratified by their behavioral profiles and sex [153]. In the same model, we recently described deregulated expression of some ERVs and ERV-related genes both in the hippocampus and prefrontal cortex, with sex-related differences marked in the latter region. Altogether, these observations reinforced the view of tissue- and sex-specificity of ERV transcriptional profiles in preclinical models of ASD [151].

Interestingly, ADHD children also showed high expression levels of HERV-H in PBMCs, with envelope (Env) transcript levels correlating positively with inattention and hyperactivity [154]. In addition, drug-naive ADHD patients showed a reduction in HERV-H Env mRNA levels in response to the administration of methylphenidate (MPH), a commonly used drug for treating ADHD symptoms. Specifically, the reduction of HERV-H expression levels H was revealed after only 1 week of MPH therapy, with a further decrease at 24 weeks of treatments in parallel with the improvement in symptoms, indicating HERV-H as a predictive marker of the response to MPH therapy [155].

As a whole, the results of deregulated HERV expression designate HERV activation as a common feature shared by several risk factors for ASD. In addition, the vicious circle made by HERV upregulation and inflammatory mediators could lead to a continuous stimulation of the immune system that could, in turn, sustain the onset and/or progression of this condition [12,156].

In addition to mRNA, a recent study performed in a small group of Sardinia patients investigated the anti-HERV-W, -K, and -H antibody response against env proteins to answer the question of whether the anti-HERV profile of ASD children differs from that of their mothers and matched healthy child/mother pairs, or whether it relates to ASD clinical severity. Interestingly, we found a new scenario supporting the hypothesis of a possible specific role of some anti-HERV responses in the modulation of peculiar symptoms of ASD. In particular, we observed that children and adolescents with ASD with the highest adaptive global functioning are those with the lowest anti-HERV-W env levels [18].

The link between anti-HERV response and ASD social dysfunction may be intriguing since, despite little is known about their function, HERV proteins are expressed in normal human brains [157] and can be recognized as “self” and immune tolerated. However, perhaps owing to their similarity with exogenous retroviral proteins, HERV antigens can trigger immune responses. In the ASD brain, chronic microglia activation [158] can induce HERV-W and -K env overexpression [159] and the subsequent activation of other immune branches, which would maintain the immune-mediated damage of the ASD brain tissues [160]. Besides microglial chronic activation, HERVs can be upregulated by epigenetic signals activated by stressful or traumatic experiences, such as social maladaptation, in children and adolescents with ASD [161].

### 3.2. Human Endogenous Retroviruses: Therapeutic Implications in Autoimmune and Neurological Diseases

Starting from the recognized involvement of envelope protein pathogenic HERV-W (pHERV-W) ENV (formerly multiple sclerosis-associated retrovirus [MSRV]-ENV) in the pathophysiological process of multiple sclerosis (MS) and type 1 diabetes (T1D) [162,163], the monoclonal antibody (mAb) temelimab, also known as GNbAC1, which selectively binds with high affinity to the extracellular domain of the pHERV-W Env, was selected for clinical development [162]. Phase I, Phase II-a, and Phase II-b trials demonstrated its safety and effectiveness for MS patients with no adverse events and provided encouraging results about its neuroprotective and regenerative effects [162,164,165]. Also, during trials with T1D patients, the antibody was well tolerated and reduced the events of hypoglycemia and the levels of anti-insulin autoantibodies after the first period of treatment [166,167].

In addition to the antibody-based immunotherapy targeting HERV ENV, the use of antiretroviral drugs has been tested by using in vitro studies up to conducting clinical trials. Concerning MS, the rationale for using these drugs is that patients with HIV treated with antiretroviral drugs show a lower risk of developing MS than the non-infected, healthy population, suggesting that the antiretroviral treatment may reduce the risk of evolving MS also acting on HERV expression [168,169]. Starting from the different reports describing HERV-K reactivation in ALS patients, such as elevated retroviral enzyme reverse transcriptase activity in the serum [170] and high expression of HERV-K expression in postmortem ALS patients’ brain tissue [14], the use of antiretroviral drugs has also been proposed in this setting. As such, in a Phase 2a, 24-week open-label study, the administration of antiretroviral combination therapy (Abacavir 600 mg, lamivudine 300 mg, dolutegravir 50 mg) was not related to any severe adverse events. Common adverse events comprised upper respiratory tract infection, urinary tract infection, nausea, diarrhea, and headache. Of note, a high percentage of patients were classified as “responsive” to treatment, reinforcing the role of HERV-K in the clinical course of the disease [11,171].

In the context of ASD, an in vitro proof of concept study using peripheral blood mononuclear cells (PBMCs) from ASD children and their parents compared to age- and sex-matched healthy controls was conducted. As such, lymphocytes were exposed to stimulating factors (Interleukin-2/Phytohaemagglutinin) or drugs, such as the antiepileptic drug valproic acid (VPA) and the antiretroviral drug efavirenz (EFV) with the intent to investigate whether the expression level of HERVs and cytokines could be modulated. Lymphocytes from ASD children and their mothers share intrinsic responsiveness to stimulating factors and VPA in expressing HERVs and cytokines. EFV specifically restored the HERV activity with a concomitant modulation of cytokines, in particular lowering the pro-inflammatory ones while maintaining high regulatory ones. This evidence provided new insights into the potential role of HERVs as biomarkers of ASD, raising the chance of using HERV expression as a therapeutic target for a multimodal approach to patient care [172].

## 4. Conclusions

In this review, we emphasize that the complex pathogenic processes that contribute to ASD can be refocused on an additional endophenotypic trait, i.e., the HERVs, that is reflective of inherited mechanisms but can be (re)activated by external newly occurring events, such as infections or stressful events. We think HERVs possess the criteria for being considered as a candidate endophenotype in ASD. As extensively discussed above, HERV genes are common in the general population, although the expression of selected HERV sub-families is over-represented in patients with ASD and their relatives; furthermore, the expression of certain HERVs could be considered as ASD-associated biological traits.

Since ASD is a heterogeneous group of neurodevelopmental disorders, a single determinant alone could be not enough to account for the complexity. HERV/cytokines expression, as well as the antibody response against HERV proteins, could be considered in a set of biomarkers, easily detectable in blood, and potentially useful for early diagnosis and subsequent intervention. This new field of research requires a renewed focus on study designs with measurement of familial co-variation of HERVs with selected ASD trait dimensions, which would possibly include family trio, sibling, and mono-/dizygotic twin studies. Moreover, it is important to highlight that for ASD children, no pharmacological treatments are available for the core symptoms, and the findings describing the downregulation of HERV expression, specifically HERV-H, by using in vitro antiretroviral treatments raises the chance of using HERV expression as a therapeutic target for a multimodal approach to patient care. As a whole, the evidence supports the hypothesis of the involvement of HERVs in ASD; however, further efforts are needed to achieve definitive proof of these elements as cofactors of neurodevelopmental disorders. These efforts could also be carried out using Artificial Intelligence (AI), a powerful tool that is proving useful in speeding up our understanding of ASD and the relationship between genetic factors and environmental triggers. Also, in neuroimaging studies, it has been shown that AI can improve the analysis of brain scans and detect subtle changes that are associated with ASD. Given the importance of HERVs in neurological disease and the recent discovery of their involvement in ASD, we are confident that AI will enable us to discover patterns and correlations that were previously hidden and could not be uncovered by traditional methods. Thanks to this amazing tool, researchers are now capable of analyzing complex data from multiple sources, including genetic, transcriptomic, and clinical datasets. By analyzing genetic and environmental factors, AI could help identify individuals at higher risk and predict how variations in HERV activity might influence disease outcomes. Using machine learning techniques, we can analyze gene expression data, including HERV RNA levels, to identify expression patterns associated with ASD. This approach can provide a deeper understanding of how HERVs may affect neurodevelopment. In addition, by analyzing large-scale biological data, we can facilitate the identification of other potential biomarkers for ASD. These biomarkers could improve early diagnosis and monitoring of disease progression. Finally, experiments in animal models could be useful for studies focusing on the molecular mechanisms underlying ASD, with the aim of investigating the further implication of HERVs in autism and developing new therapeutic strategies.

## References

[1] Lord C., Elsabbagh M., Baird G., Veenstra-Vanderweele J. (2018). Autism Spectrum Disorder. Lancet.

[2] Meltzer A., Van De Water J. (2017). The Role of the Immune System in Autism Spectrum Disorder. Neuropsychopharmacology.

[3] Bai D., Yip B.H.K., Windham G.C., Sourander A., Francis R., Yoffe R., Glasson E., Mahjani B., Suominen A., Leonard H. (2019). Association of Genetic and Environmental Factors with Autism in a 5-Country Cohort. JAMA Psychiatry.

[4] Jensen A.R., Lane A.L., Werner B.A., McLees S.E., Fletcher T.S., Frye R.E. (2022). Modern Biomarkers for Autism Spectrum Disorder: Future Directions. Mol. Diagn. Ther..

[5] Nisar S., Haris M. (2023). Correction to: Neuroimaging Genetics Approaches to Identify New Biomarkers for the Early Diagnosis of Autism Spectrum Disorder. Mol. Psychiatry.

[6] Iacono W.G. (2018). Endophenotypes in Psychiatric Disease: Prospects and Challenges. Genome Med..

[7] Mosconi M.W., Stevens C.J., Unruh K.E., Shafer R., Elison J.T. (2023). Correction to: Endophenotype Trait Domains for Advancing Gene Discovery in Autism Spectrum Disorder. J. Neurodev. Disord..

[8] Constantino J.N., Kennon-McGill S., Weichselbaum C., Marrus N., Haider A., Glowinski A.L., Gillespie S., Klaiman C., Klin A., Jones W. (2017). Infant Viewing of Social Scenes Is under Genetic Control and Is Atypical in Autism. Nature.

[9] Esposito D., Cruciani G., Zaccaro L., Di Carlo E., Spitoni G.F., Manti F., Carducci C., Fiori E., Leuzzi V., Pascucci T. (2024). A Systematic Review on Autism and Hyperserotonemia: State-of-the-Art, Limitations, and Future Directions. Brain Sci..

[10] Kundu S., Sair H., Sherr E.H., Mukherjee P., Rohde G.K. (2024). Discovering the Gene-Brain-Behavior Link in Autism via Generative Machine Learning. Sci. Adv..

[11] Garcia-Montojo M., Fathi S., Norato G., Smith B.R., Rowe D.B., Kiernan M.C., Vucic S., Mathers S., van Eijk R.P.A., Santamaria U. (2021). Inhibition of HERV-K (HML-2) in Amyotrophic Lateral Sclerosis Patients on Antiretroviral Therapy. J. Neurol. Sci..

[12] Balestrieri E., Matteucci C., Cipriani C., Grelli S., Ricceri L., Calamandrei G., Vallebona P.S. (2019). Endogenous Retroviruses Activity as a Molecular Signature of Neurodevelopmental Disorders. Int. J. Mol. Sci..

[13] Küry P., Nath A., Créange A., Dolei A., Marche P., Gold J., Giovannoni G., Hartung H.P., Perron H. (2018). Human Endogenous Retroviruses in Neurological Diseases. Trends Mol. Med..

[14] Li W., Lee M.H., Henderson L., Tyagi R., Bachani M., Steiner J., Campanac E., Hoffman D.A., Von Geldern G., Johnson K. (2015). Human Endogenous Retrovirus-K Contributes to Motor Neuron Disease. Sci. Transl. Med..

[15] Padmanabhan Nair V., Liu H., Ciceri G., Jungverdorben J., Frishman G., Tchieu J., Cederquist G.Y., Rothenaigner I., Schorpp K., Klepper L. (2021). Activation of HERV-K(HML-2) Disrupts Cortical Patterning and Neuronal Differentiation by Increasing NTRK3. Cell Stem Cell.

[16] Johansson E.M., Bouchet D., Tamouza R., Ellul P., Morr A.S., Avignone E., Germi R., Leboyer M., Perron H., Groc L. (2020). Human Endogenous Retroviral Protein Triggers Deficit in Glutamate Synapse Maturation and Behaviors Associated with Psychosis. Sci. Adv..

[17] Balestrieri E., Arpino C., Matteucci C., Sorrentino R., Pica F., Alessandrelli R., Coniglio A., Curatolo P., Rezza G., Macciardi F. (2012). HERVs Expression in Autism Spectrum Disorders. PLoS ONE.

[18] Carta A., Manca M.A., Scoppola C., Simula E.R., Noli M., Ruberto S., Conti M., Zarbo I.R., Antonucci R., Sechi L.A. (2022). Antihuman Endogenous Retrovirus Immune Response and Adaptive Dysfunction in Autism. Biomedicines.

[19] Pizzioli E., Minutolo A., Balestrieri E., Matteucci C., Magiorkinis G., Horvat B. (2024). Crosstalk between Human Endogenous Retroviruses and Exogenous Viruses. Microbes Infect..

[20] Gholami Barzoki M., Shatizadeh Malekshahi S., Heydarifard Z., Mahmodi M.J., Soltanghoraee H. (2023). The Important Biological Roles of Syncytin-1 of Human Endogenous Retrovirus W (HERV-W) and Syncytin-2 of HERV-FRD in the Human Placenta Development. Mol. Biol. Rep..

[21] Cossu D., Tomizawa Y., Sechi L.A., Hattori N. (2023). Epstein–Barr Virus and Human Endogenous Retrovirus in Japanese Patients with Autoimmune Demyelinating Disorders. Int. J. Mol. Sci..

[22] Ruberto S., Cossu D., Sechi L.A. (2024). Correlation between Antibodies against the Pathogenic PHERV-W Envelope Protein and the Inflammatory Phase of Multiple Sclerosis. Immunology.

[23] Noli M., Meloni G., Manca P., Cossu D., Palermo M., Sechi L.A. (2021). HERV-W and Mycobacterium Avium Subspecies Paratuberculosis Are at Play in Pediatric Patients at Onset of Type 1 Diabetes. Pathogens.

[24] Dow C.T., Pierce E.S., Sechi L.A. (2024). Mycobacterium Paratuberculosis: A HERV Turn-On for Autoimmunity, Neurodegeneration, and Cancer?. Microorganisms.

[25] Niegowska M., Wajda-Cuszlag M., Stępień-Ptak G., Trojanek J., Michałkiewicz J., Szalecki M., Sechi L.A. (2019). Anti-HERV-WEnv Antibodies Are Correlated with Seroreactivity against Mycobacterium Avium Subsp. Paratuberculosis in Children and Youths at T1D Risk. Sci. Rep..

[26] Jönsson M.E., Garza R., Johansson P.A., Jakobsson J. (2020). Transposable Elements: A Common Feature of Neurodevelopmental and Neurodegenerative Disorders. Trends Genet..

[27] Jönsson M.E., Garza R., Sharma Y., Petri R., Södersten E., Johansson J.G., Johansson P.A., Atacho D.A., Pircs K., Madsen S. (2021). Activation of Endogenous Retroviruses during Brain Development Causes an Inflammatory Response. EMBO J..

[28] Kitsou K., Lagiou P., Magiorkinis G. (2023). Human Endogenous Retroviruses in Cancer: Oncogenesis Mechanisms and Clinical Implications. J. Med. Virol..

[29] Bo M., Manetti R., Biggio M.L., Sechi L.A. (2024). The Humoral Immune Response against Human Endogenous Retroviruses in Celiac Disease: A Case–Control Study. Biomedicines.

[30] Noli M., Meloni G., Ruberto S., Jasemi S., Simula E.R., Cossu D., Bo M., Palermo M., Sechi L.A. (2022). HERV-K Envelope Protein Induces Long-Lasting Production of Autoantibodies in T1DM Patients at Onset in Comparison to ZNT8 Autoantibodies. Pathogens.

[31] Arru G., Sechi E., Mariotto S., Zarbo I.R., Ferrari S., Gajofatto A., Monaco S., Deiana G.A., Bo M., Sechi L.A. (2020). Antibody Response against HERV-W in Patients with MOG-IgG Associated Disorders, Multiple Sclerosis and NMOSD. J. Neuroimmunol..

[32] Mameli G., Erre G.L., Caggiu E., Mura S., Cossu D., Bo M., Cadoni M.L., Piras A., Mundula N., Colombo E. (2017). Identification of a HERV-K Env Surface Peptide Highly Recognized in Rheumatoid Arthritis (RA) Patients: A Cross-Sectional Case–Control Study. Clin. Exp. Immunol..

[33] Arru G., Mameli G., Deiana G.A., Rassu A.L., Piredda R., Sechi E., Caggiu E., Bo M., Nako E., Urso D. (2018). Humoral Immunity Response to Human Endogenous Retroviruses K/W Differentiates between Amyotrophic Lateral Sclerosis and Other Neurological Diseases. Eur. J. Neurol..

[34] Jasemi S., Erre G.L., Cadoni M.L., Bo M., Sechi L.A. (2021). Humoral Response to Microbial Biomarkers in Rheumatoid Arthritis Patients. J. Clin. Med..

[35] Wang J., Ren M., Yu J., Hu M., Wang X., Ma W., Jiang X., Cui J. (2022). Single-Cell RNA Sequencing Highlights the Functional Role of Human Endogenous Retroviruses in Gallbladder Cancer. EBioMedicine.

[36] La Ferlita A., Nigita G., Tsyba L., Palamarchuk A., Alaimo S., Pulvirenti A., Balatti V., Rassenti L., Tsichlis P.N., Kipps T. (2023). Expression Signature of Human Endogenous Retroviruses in Chronic Lymphocytic Leukemia. Proc. Natl. Acad. Sci. USA.

[37] Urru S.A.M., Antonelli A., Sechi G.M. (2020). Prevalence of Multiple Sclerosis in Sardinia: A Systematic Cross-Sectional Multi-Source Survey. Mult. Scler. J..

[38] Puthenparampil M., Perini P., Bergamaschi R., Capobianco M., Filippi M., Gallo P. (2022). Multiple Sclerosis Epidemiological Trends in Italy Highlight the Environmental Risk Factors. J. Neurol..

[39] Sotgiu S., Onida I., Magli G., Castiglia P., Conti M., Nuvoli A., Carta A., Festa S., Dessì V., Doneddu P.E. (2021). Juvenile Chronic Inflammatory Demyelinating Polyneuropathy Epidemiology in Sardinia, Insular Italy. Neuropediatrics.

[40] Dell’Avvento S., Sotgiu M.A., Manca S., Sotgiu G., Sotgiu S. (2016). Epidemiology of Multiple Sclerosis in the Pediatric Population of Sardinia, Italy. Eur. J. Pediatr..

[41] Storm C.S., Kia D.A., Almramhi M., Wood N.W. (2020). Using Mendelian Randomization to Understand and Develop Treatments for Neurodegenerative Disease. Brain Commun..

[42] American Psychiatric Association (2022). Diagnostic and Statistical Manual of Mental Disorders, Fifth Edition, Text Revision (DSM-5-TR).

[43] Hirota T., King B.H. (2023). Autism Spectrum Disorder. JAMA.

[44] Maenner M.J., Warren Z., Williams A.R., Amoakohene E., Bakian A.V., Bilder D.A., Durkin M.S., Fitzgerald R.T., Furnier S.M., Hughes M.M. (2023). Prevalence and Characteristics of Autism Spectrum Disorder Among Children Aged 8 Years—Autism and Developmental Disabilities Monitoring Network, 11 Sites, United States, 2020. MMWR. Surveill. Summ..

[45] Huda E., Hawker P., Cibralic S., John J.R., Hussain A., Diaz A.M., Eapen V. (2024). Screening Tools for Autism in Culturally and Linguistically Diverse Paediatric Populations: A Systematic Review. BMC Pediatr..

[46] Hyman S.L., Levy S.E., Myers S.M., Kuo D.Z., Apkon S., Davidson L.F., Ellerbeck K.A., Foster J.E.A., Noritz G.H., Leppert M.O. (2020). Identification, Evaluation, and Management of Children with Autism Spectrum Disorder. Pediatrics.

[47] Lord C., Charman T., Havdahl A., Carbone P., Anagnostou E., Boyd B., Carr T., de Vries P.J., Dissanayake C., Divan G. (2022). The Lancet Commission on the Future of Care and Clinical Research in Autism. Lancet.

[48] Zack D.S., Carroll B., Magallanes A., Bordes Edgar V. (2024). Take a Closer Look: Considerations for Autism Spectrum Disorder Assessment in Female Children and Adolescents. J. Pediatr. Health Care.

[49] Cruz S., Zubizarreta S.C.-P., Costa A.D., Araújo R., Martinho J., Tubío-Fungueiriño M., Sampaio A., Cruz R., Carracedo A., Fernández-Prieto M. (2024). Is There a Bias Towards Males in the Diagnosis of Autism? A Systematic Review and Meta-Analysis. Neuropsychol. Rev..

[50] Rippon G. (2024). Differently Different?: A Commentary on the Emerging Social Cognitive Neuroscience of Female Autism. Biol. Sex Differ..

[51] dos Santos C.L., Barreto I.I., Floriano I., Tristão L.S., Silvinato A., Bernardo W.M. (2024). Screening and Diagnostic Tools for Autism Spectrum Disorder: Systematic Review and Meta-Analysis. Clinics..

[52] Yu Y., Ozonoff S., Miller M. (2024). Assessment of Autism Spectrum Disorder. Assessment.

[53] Lord C., Rutter M., Le Couteur A. (1994). Autism Diagnostic Interview-Revised: A Revised Version of a Diagnostic Interview for Caregivers of Individuals with Possible Pervasive Developmental Disorders. J. Autism Dev. Disord..

[54] Schopler E., Reichler R.J., DeVellis R.F., Daly K. (2016). Childhood Autism Rating Scale. PsycTESTS Dataset.

[55] McCrimmon A., Rostad K. (2014). Test Review: Autism Diagnostic Observation Schedule, Second Edition (ADOS-2) Manual (Part II): Toddler Module. J. Psychoeduc. Assess..

[56] Shulman C., Esler A., Morrier M.J., Rice C.E. (2020). Diagnosis of Autism Spectrum Disorder Across the Lifespan. Child Adolesc. Psychiatr. Clin. N. Am..

[57] Loubersac J., Michelon C., Ferrando L., Picot M.-C., Baghdadli A. (2023). Predictors of an Earlier Diagnosis of Autism Spectrum Disorder in Children and Adolescents: A Systematic Review (1987–2017). Eur. Child Adolesc. Psychiatry.

[58] Rosen N.E., Lord C., Volkmar F.R. (2021). The Diagnosis of Autism: From Kanner to DSM-III to DSM-5 and Beyond. J. Autism Dev. Disord..

[59] Qin L., Wang H., Ning W., Cui M., Wang Q. (2024). New Advances in the Diagnosis and Treatment of Autism Spectrum Disorders. Eur. J. Med. Res..

[60] Thurm A., Powell E.M., Neul J.L., Wagner A., Zwaigenbaum L. (2018). Loss of Skills and Onset Patterns in Neurodevelopmental Disorders: Understanding the Neurobiological Mechanisms. Autism Res..

[61] Tanner A., Dounavi K. (2021). The Emergence of Autism Symptoms Prior to 18 Months of Age: A Systematic Literature Review. J. Autism Dev. Disord..

[62] Baron-Cohen S., Allen J., Gillberg C. (1992). Can Autism Be Detected at 18 Months?. Br. J. Psychiatry.

[63] Hadders-Algra M. (2022). Emerging Signs of Autism Spectrum Disorder in Infancy: Putative Neural Substrate. Dev. Med. Child Neurol..

[64] Baranek G.T., Watson L.R., Boyd B.A., Poe M.D., David F.J., McGuire L. (2013). Hyporesponsiveness to Social and Nonsocial Sensory Stimuli in Children with Autism, Children with Developmental Delays, and Typically Developing Children. Dev. Psychopathol..

[65] Masjedi N., Clarke E.B., Lord C. (2024). Development of Restricted and Repetitive Behaviors from 2–19: Stability and Change in Repetitive Sensorimotor, Insistence on Sameness, and Verbal Behaviors in a Longitudinal Study of Autism. J. Autism Dev. Disord..

[66] Sicherman N., Charite J., Eyal G., Janecka M., Loewenstein G., Law K., Lipkin P.H., Marvin A.R., Buxbaum J.D. (2021). Clinical Signs Associated with Earlier Diagnosis of Children with Autism Spectrum Disorder. BMC Pediatr..

[67] Pires J.F., Grattão C.C., Gomes R.M.R. (2024). The Challenges for Early Intervention and Its Effects on the Prognosis of Autism Spectrum Disorder: A Systematic Review. Dement. Neuropsychol..

[68] Waizbard-Bartov E., Fein D., Lord C., Amaral D.G. (2023). Autism Severity and Its Relationship to Disability. Autism Res..

[69] Schaeffer J., Abd El-Raziq M., Castroviejo E., Durrleman S., Ferré S., Grama I., Hendriks P., Kissine M., Manenti M., Marinis T. (2023). Language in Autism: Domains, Profiles and Co-Occurring Conditions. J. Neural Transm..

[70] Denisova K. (2024). Neurobiology of Cognitive Abilities in Early Childhood Autism. JCPP Adv..

[71] Carta A., Fucà E., Guerrera S., Napoli E., Valeri G., Vicari S. (2020). Characterization of Clinical Manifestations in the Co-Occurring Phenotype of Attention Deficit/Hyperactivity Disorder and Autism Spectrum Disorder. Front. Psychol..

[72] Lai M.-C., Kassee C., Besney R., Bonato S., Hull L., Mandy W., Szatmari P., Ameis S.H. (2019). Prevalence of Co-Occurring Mental Health Diagnoses in the Autism Population: A Systematic Review and Meta-Analysis. Lancet Psychiatry.

[73] Kaye A.D., Allen K.E., Smith III V.S., Tong V.T., Mire V.E., Nguyen H., Lee Z., Kouri M., Jean Baptiste C., Mosieri C.N. (2024). Emerging Treatments and Therapies for Autism Spectrum Disorder: A Narrative Review. Cureus.

[74] Narzisi A., Posada M., Barbieri F., Chericoni N., Ciuffolini D., Pinzino M., Romano R., Scattoni M.L., Tancredi R., Calderoni S. (2020). Prevalence of Autism Spectrum Disorder in a Large Italian Catchment Area: A School-Based Population Study within the ASDEU Project. Epidemiol. Psychiatr. Sci..

[75] Sotgiu S., Manca S., Gagliano A., Minutolo A., Melis M.C., Pisuttu G., Scoppola C., Bolognesi E., Clerici M., Guerini F.R. (2020). Immune Regulation of Neurodevelopment at the Mother–Foetus Interface: The Case of Autism. Clin. Transl. Immunol..

[76] Schneider J.S., Kidd S.K., Anderson D.W. (2013). Influence of Developmental Lead Exposure on Expression of DNA Methyltransferases and Methyl Cytosine-Binding Proteins in Hippocampus. Toxicol. Lett..

[77] Pugsley K., Scherer S.W., Bellgrove M.A., Hawi Z. (2022). Environmental Exposures Associated with Elevated Risk for Autism Spectrum Disorder May Augment the Burden of Deleterious de Novo Mutations among Probands. Mol. Psychiatry.

[78] Kaur I., Behl T., Aleya L., Rahman M.H., Kumar A., Arora S., Akter R. (2021). Role of Metallic Pollutants in Neurodegeneration: Effects of Aluminum, Lead, Mercury, and Arsenic in Mediating Brain Impairment Events and Autism Spectrum Disorder. Environ. Sci. Pollut. Res..

[79] Baj J., Flieger W., Flieger M., Forma A., Sitarz E., Skórzyńska-Dziduszko K., Grochowski C., Maciejewski R., Karakuła-Juchnowicz H. (2021). Autism Spectrum Disorder: Trace Elements Imbalances and the Pathogenesis and Severity of Autistic Symptoms. Neurosci. Biobehav. Rev..

[80] Yousefi B., Eslami M., Ghasemian A., Kokhaei P., Sadeghnejhad A. (2019). Probiotics Can Really Cure an Autoimmune Disease?. Gene Rep..

[81] Eslami M., Bahar A., Hemati M., Rasouli Nejad Z., Mehranfar F., Karami S., Kobyliak N.M., Yousefi B. (2021). Dietary Pattern, Colonic Microbiota and Immunometabolism Interaction: New Frontiers for Diabetes Mellitus and Related Disorders. Diabet. Med..

[82] Madra M., Ringel R., Margolis K.G. (2020). Gastrointestinal Issues and Autism Spectrum Disorder. Child Adolesc. Psychiatr. Clin. N. Am..

[83] Taniya M.A., Chung H.J., Al Mamun A., Alam S., Aziz M.A., Emon N.U., Islam M.M., Hong S.T.S., Podder B.R., Ara Mimi A. (2022). Role of Gut Microbiome in Autism Spectrum Disorder and Its Therapeutic Regulation. Front. Cell. Infect. Microbiol..

[84] Wang W., Fu P. (2023). Gut Microbiota Analysis and In Silico Biomarker Detection of Children with Autism Spectrum Disorder across Cohorts. Microorganisms.

[85] Wan Y., Zuo T., Xu Z., Zhang F., Zhan H., Chan D., Leung T.F., Yeoh Y.K., Chan F.K.L., Chan R. (2022). Underdevelopment of the Gut Microbiota and Bacteria Species as Non-Invasive Markers of Prediction in Children with Autism Spectrum Disorder. Gut.

[86] Jacobson A., Yang D., Vella M., Chiu I.M. (2021). The Intestinal Neuro-Immune Axis: Crosstalk between Neurons, Immune Cells, and Microbes. Mucosal Immunol..

[87] Roman P., Rueda-Ruzafa L., Cardona D., Cortes-Rodríguez A. (2018). Gut-Brain Axis in the Executive Function of Austism Spectrum Disorder. Behav. Pharmacol..

[88] Yano J.M., Yu K., Donaldson G.P., Shastri G.G., Ann P., Ma L., Nagler C.R., Ismagilov R.F., Mazmanian S.K., Hsiao E.Y. (2015). Indigenous Bacteria from the Gut Microbiota Regulate Host Serotonin Biosynthesis. Cell.

[89] Goines P., Van De Water J. (2010). The Immune System’s Role in the Biology of Autism. Curr. Opin. Neurol..

[90] Persico A.M., Napolioni V. (2013). Urinary P-Cresol in Autism Spectrum Disorder. Neurotoxicol. Teratol..

[91] Passmore I.J., Letertre M.P.M., Preston M.D., Bianconi I., Harrison M.A., Nasher F., Kaur H., Hong H.A., Baines S.D., Cutting S.M. (2018). Para-Cresol Production by Clostridium Difficile Affects Microbial Diversity and Membrane Integrity of Gram-Negative Bacteria. PLoS Pathog..

[92] Williams B.L., Hornig M., Parekh T., Ian Lipkin W. (2012). Application of Novel PCR-Based Methods for Detection, Quantitation, and Phylogenetic Characterization of Sutterella Species in Intestinal Biopsy Samples from Children with Autism and Gastrointestinal Disturbances. mBio.

[93] Ding H.T., Taur Y., Walkup J.T. (2017). Gut Microbiota and Autism: Key Concepts and Findings. J. Autism Dev. Disord..

[94] Kang D.W., Park J.G., Ilhan Z.E., Wallstrom G., LaBaer J., Adams J.B., Krajmalnik-Brown R. (2013). Reduced Incidence of Prevotella and Other Fermenters in Intestinal Microflora of Autistic Children. PLoS ONE.

[95] Li Z., Liu S., Liu F., Dai N., Liang R., Lv S., Bao L. (2023). Gut Microbiota and Autism Spectrum Disorders: A Bidirectional Mendelian Randomization Study. Front. Cell. Infect. Microbiol..

[96] Yang Y., Tian J., Yang B. (2018). Targeting Gut Microbiome: A Novel and Potential Therapy for Autism. Life Sci..

[97] Shaaban S.Y., El Gendy Y.G., Mehanna N.S., El-Senousy W.M., El-Feki H.S.A., Saad K., El-Asheer O.M. (2018). The Role of Probiotics in Children with Autism Spectrum Disorder: A Prospective, Open-Label Study. Nutr. Neurosci..

[98] Wang X., Yang J., Zhang H., Yu J., Yao Z. (2019). Oral Probiotic Administration during Pregnancy Prevents Autism-Related Behaviors in Offspring Induced by Maternal Immune Activation via Anti-Inflammation in Mice. Autism Res..

[99] Tioleco N., Silberman A.E., Stratigos K., Banerjee-Basu S., Spann M.N., Whitaker A.H., Turner J.B. (2021). Prenatal Maternal Infection and Risk for Autism in Offspring: A Meta-analysis. Autism Res..

[100] Brynge M., Sjöqvist H., Gardner R.M., Lee B.K., Dalman C., Karlsson H. (2022). Maternal Infection during Pregnancy and Likelihood of Autism and Intellectual Disability in Children in Sweden: A Negative Control and Sibling Comparison Cohort Study. Lancet Psychiatry.

[101] Christensen J., Grønborg T.K., Sørensen M.J., Schendel D., Parner E.T., Pedersen L.H., Vestergaard M. (2013). Prenatal Valproate Exposure and Risk of Autism Spectrum Disorders and Childhood Autism. JAMA.

[102] Sanders S.J., He X., Willsey A.J., Ercan-Sencicek A.G., Samocha K.E., Cicek A.E., Murtha M.T., Bal V.H., Bishop S.L., Dong S. (2015). Insights into Autism Spectrum Disorder Genomic Architecture and Biology from 71 Risk Loci. Neuron.

[103] Marshall C.R., Noor A., Vincent J.B., Lionel A.C., Feuk L., Skaug J., Shago M., Moessner R., Pinto D., Ren Y. (2008). Structural Variation of Chromosomes in Autism Spectrum Disorder. Am. J. Hum. Genet..

[104] Sandin S., Lichtenstein P., Kuja-Halkola R., Larsson H., Hultman C.M., Reichenberg A. (2014). The Familial Risk of Autism. JAMA.

[105] Modabbernia A., Velthorst E., Reichenberg A. (2017). Environmental Risk Factors for Autism: An Evidence-Based Review of Systematic Reviews and Meta-Analyses. Mol. Autism.

[106] Grove J., Ripke S., Als T.D., Mattheisen M., Walters R.K., Won H., Pallesen J., Agerbo E., Andreassen O.A., Anney R. (2019). Identification of Common Genetic Risk Variants for Autism Spectrum Disorder. Nat. Genet..

[107] Vorstman J.A.S., Parr J.R., Moreno-De-Luca D., Anney R.J.L., Nurnberger J.I., Hallmayer J.F. (2017). Autism Genetics: Opportunities and Challenges for Clinical Translation. Nat. Rev. Genet..

[108] Persico A.M., Napolioni V. (2013). Autism Genetics. Behav. Brain Res..

[109] Kim J.Y., Son M.J., Son C.Y., Radua J., Eisenhut M., Gressier F., Koyanagi A., Carvalho A.F., Stubbs B., Solmi M. (2019). Environmental Risk Factors and Biomarkers for Autism Spectrum Disorder: An Umbrella Review of the Evidence. Lancet Psychiatry.

[110] Abrahams B.S., Geschwind D.H. (2008). Advances in Autism Genetics: On the Threshold of a New Neurobiology. Nat. Rev. Genet..

[111] Loke Y.J., Hannan A.J., Craig J.M. (2015). The Role of Epigenetic Change in Autism Spectrum Disorders. Front. Neurol..

[112] Guerini F.R., Bolognesi E., Manca S., Sotgiu S., Zanzottera M., Agliardi C., Usai S., Clerici M. (2009). Family-Based Transmission Analysis of HLA Genetic Markers in Sardinian Children with Autistic Spectrum Disorders. Hum. Immunol..

[113] Guerini F.R., Bolognesi E., Chiappedi M., De Silvestri A., Ghezzo A., Zanette M., Rusconi B., Manca S., Sotgiu S., Agliardi C. (2011). HLA Polymorphisms in Italian Children with Autism Spectrum Disorders: Results of a Family Based Linkage Study. J. Neuroimmunol..

[114] Fu B., Zhou Y., Ni X., Tong X., Xu X., Dong Z., Sun R., Tian Z., Wei H. (2017). Natural Killer Cells Promote Fetal Development through the Secretion of Growth-Promoting Factors. Immunity.

[115] Cook K.D., Waggoner S.N., Whitmire J.K. (2014). NK Cells and Their Ability to Modulate T Cells during Virus Infections. Crit. Rev. Immunol..

[116] Guerini F.R., Bolognesi E., Chiappedi M., Manca S., Ghezzo A., Agliardi C., Zanette M., Littera R., Carcassi C., Sotgiu S. (2014). Activating KIR Molecules and Their Cognate Ligands Prevail in Children with a Diagnosis of ASD and in Their Mothers. Brain Behav. Immun..

[117] Guerini F.R., Bolognesi E., Chiappedi M., Ripamonti E., Ghezzo A., Zanette M., Sotgiu S., Mensi M.M., Carta A., Canevini M.P. (2018). HLA-G Coding Region Polymorphism Is Skewed in Autistic Spectrum Disorders. Brain Behav. Immun..

[118] Guerini F.R., Bolognesi E., Chiappedi M., Ghezzo A., Manca S., Zanette M., Sotgiu S., Mensi M.M., Zanzottera M., Agliardi C. (2018). HLA-G∗14bp Insertion and the KIR2DS1-HLAC2 Complex Impact on Behavioral Impairment in Children with Autism Spectrum Disorders. Neuroscience.

[119] Desoky T., Hassan M.H., Fayed H., Sakhr H.M. (2017). Biochemical Assessments of Thyroid Profile, Serum 25-Hydroxycholecalciferol and Cluster of Differentiation 5 Expression Levels among Children with Autism. Neuropsychiatr. Dis. Treat..

[120] Guerini F.R., Bolognesi E., Chiappedi M., Mensi M.M., Fumagalli O., Rogantini C., Zanzottera M., Ghezzo A., Zanette M., Agliardi C. (2020). Vitamin D Receptor Polymorphisms Associated with Autism Spectrum Disorder. Autism Res..

[121] Bolognesi E., Guerini F.R., Sotgiu S., Chiappedi M., Carta A., Mensi M.M., Agliardi C., Zanzottera M., Clerici M. (2022). GC1f Vitamin D Binding Protein Isoform as a Marker of Severity in Autism Spectrum Disorders. Nutrients.

[122] Nudel R., Thompson W.K., Børglum A.D., Hougaard D.M., Mortensen P.B., Werge T., Nordentoft M., Benros M.E. (2022). Maternal Pregnancy-Related Infections and Autism Spectrum Disorder—The Genetic Perspective. Transl. Psychiatry.

[123] Howlin P., Magiati I., Charman T. (2009). Systematic Review of Early Intensive Behavioral Interventions for Children with Autism. Am. J. Intellect. Dev. Disabil..

[124] Schreibman L., Dawson G., Stahmer A.C., Landa R., Rogers S.J., McGee G.G., Kasari C., Ingersoll B., Kaiser A.P., Bruinsma Y. (2015). Naturalistic Developmental Behavioral Interventions: Empirically Validated Treatments for Autism Spectrum Disorder. J. Autism Dev. Disord..

[125] Mesibov G.B., Shea V. (2010). The TEACCH Program in the Era of Evidence-Based Practice. J. Autism Dev. Disord..

[126] Prizant B.M., Wetherby A.M., Rubin E., Laurent A.C., Rydell P.J. (2006). The SCERTS Model: A Comprehensive Educational Approach for Children with Autism Spectrum Disorders.

[127] Kasari C., Gulsrud A., Paparella T., Hellemann G., Berry K. (2015). Randomized Comparative Efficacy Study of Parent-Mediated Interventions for Toddlers with Autism. J. Consult. Clin. Psychol..

[128] Valeri G., Casula L., Menghini D., Amendola F.A., Napoli E., Pasqualetti P., Vicari S. (2020). Cooperative Parent-Mediated Therapy for Italian Preschool Children with Autism Spectrum Disorder: A Randomized Controlled Trial. Eur. Child Adolesc. Psychiatry.

[129] Conrad C.E., Rimestad M.L., Rohde J.F., Petersen B.H., Korfitsen C.B., Tarp S., Cantio C., Lauritsen M.B., Händel M.N. (2021). Parent-Mediated Interventions for Children and Adolescents with Autism Spectrum Disorders: A Systematic Review and Meta-Analysis. Front. Psychiatry.

[130] McPheeters M.L., Warren Z., Sathe N., Bruzek J.L., Krishnaswami S., Jerome R.N., Veenstra-VanderWeele J. (2011). A Systematic Review of Medical Treatments for Children with Autism Spectrum Disorders. Pediatrics.

[131] Hollander E., Phillips A.T., Yeh C.-C. (2003). Targeted Treatments for Symptom Domains in Child and Adolescent Autism. Lancet.

[132] Kolevzon A., Mathewson K.A., Hollander E. (2006). Selective Serotonin Reuptake Inhibitors in Autism. J. Clin. Psychiatry.

[133] Handen B.L., Johnson C.R., Lubetsky M. (2000). Efficacy of Methylphenidate among Children with Autism and Symptoms of Attention-Deficit Hyperactivity Disorder. J. Autism Dev. Disord..

[134] Sturman N., Deckx L., van Driel M.L. (2017). Methylphenidate for Children and Adolescents with Autism Spectrum Disorder. Cochrane Database Syst. Rev..

[135] Ecker C., Schmeisser M.J., Loth E., Murphy D.G. (2017). Neuroanatomy and Neuropathology of Autism Spectrum Disorder in Humans. Adv. Anat. Embryol. Cell Biol..

[136] Loth E., Charman T., Mason L., Tillmann J., Jones E.J.H., Wooldridge C., Ahmad J., Auyeung B., Brogna C., Ambrosino S. (2017). The EU-AIMS Longitudinal European Autism Project (LEAP): Design and Methodologies to Identify and Validate Stratification Biomarkers for Autism Spectrum Disorders. Mol. Autism.

[137] Salpekar J.A., Scahill L. (2024). Psychopharmacology Management in Autism Spectrum Disorder. Pediatr. Clin. N. Am..

[138] Balestrieri E., Cipriani C., Matteucci C., Capodicasa N., Pilika A., Korca I., Sorrentino R., Argaw-Denboba A., Bucci I., Miele M.T. (2016). Transcriptional Activity of Human Endogenous Retrovirus in Albanian Children with Autism Spectrum Disorders. New Microbiol..

[139] Heidmann O., Béguin A., Paternina J., Berthier R., Deloger M., Bawa O., Heidmann T. (2017). HEMO, an Ancestral Endogenous Retroviral Envelope Protein Shed in the Blood of Pregnant Women and Expressed in Pluripotent Stem Cells and Tumors. Proc. Natl. Acad. Sci. USA.

[140] Balestrieri E., Cipriani C., Matteucci C., Benvenuto A., Coniglio A., Argaw-Denboba A., Toschi N., Bucci I., Miele M.T., Grelli S. (2019). Children with Autism Spectrum Disorder and Their Mothers Share Abnormal Expression of Selected Endogenous Retroviruses Families and Cytokines. Front. Immunol..

[141] Tovo P.-A., Davico C., Marcotulli D., Vitiello B., Daprà V., Calvi C., Montanari P., Carpino A., Galliano I., Bergallo M. (2022). Enhanced Expression of Human Endogenous Retroviruses, TRIM28 and SETDB1 in Autism Spectrum Disorder. Int. J. Mol. Sci..

[142] Cipriani C., Ricceri L., Matteucci C., De Felice A., Tartaglione A.M., Argaw-Denboba A., Pica F., Grelli S., Calamandrei G., Sinibaldi Vallebona P. (2018). High Expression of Endogenous Retroviruses from Intrauterine Life to Adulthood in Two Mouse Models of Autism Spectrum Disorders. Sci. Rep..

[143] Tartaglione A.M., Cipriani C., Chiarotti F., Perrone B., Balestrieri E., Matteucci C., Sinibaldi-Vallebona P., Calamandrei G., Ricceri L. (2019). Early Behavioral Alterations and Increased Expression of Endogenous Retroviruses Are Inherited Across Generations in Mice Prenatally Exposed to Valproic Acid. Mol. Neurobiol..

[144] Meyza K.Z., Blanchard D.C. (2017). The BTBR Mouse Model of Idiopathic Autism—Current View on Mechanisms. Neurosci. Biobehav. Rev..

[145] Meyza K.Z., Defensor E.B., Jensen A.L., Corley M.J., Pearson B.L., Pobbe R.L.H., Bolivar V.J., Blanchard D.C., Blanchard R.J. (2013). The BTBR T+tf/J Mouse Model for Autism Spectrum Disorders-in Search of Biomarkers. Behav. Brain Res..

[146] McFarlane H.G., Kusek G.K., Yang M., Phoenix J.L., Bolivar V.J., Crawley J.N. (2008). Autism-like Behavioral Phenotypes in BTBR T+tf/J Mice. Genes Brain Behav..

[147] Scattoni M.L., Ricceri L., Crawley J.N. (2011). Unusual Repertoire of Vocalizations in Adult BTBR T+tf/J Mice during Three Types of Social Encounters. Genes Brain Behav..

[148] Ellegood J., Crawley J.N. (2015). Behavioral and Neuroanatomical Phenotypes in Mouse Models of Autism. Neurotherapeutics.

[149] Kataoka S., Takuma K., Hara Y., Maeda Y., Ago Y., Matsuda T. (2013). Autism-like Behaviours with Transient Histone Hyperacetylation in Mice Treated Prenatally with Valproic Acid. Int. J. Neuropsychopharmacol..

[150] Lin C.W., Ellegood J., Tamada K., Miura I., Konda M., Takeshita K., Atarashi K., Lerch J.P., Wakana S., McHugh T.J. (2023). An Old Model with New Insights: Endogenous Retroviruses Drive the Evolvement toward ASD Susceptibility and Hijack Transcription Machinery during Development. Mol. Psychiatry.

[151] Cipriani C., Tartaglione A.M., Giudice M., D’Avorio E., Petrone V., Toschi N., Chiarotti F., Miele M.T., Calamandrei G., Garaci E. (2022). Differential Expression of Endogenous Retroviruses and Inflammatory Mediators in Female and Male Offspring in a Mouse Model of Maternal Immune Activation. Int. J. Mol. Sci..

[152] Holt A.C., Medzhitov R., Flavell R.A., Alexopoulou L. (2001). Recognition of Double-Stranded RNA and Activation of NF-KappaB by Toll-like Receptor 3. Nature.

[153] Herrero F., Mueller F.S., Gruchot J., Küry P., Weber-Stadlbauer U., Meyer U. (2023). Susceptibility and Resilience to Maternal Immune Activation Are Associated with Differential Expression of Endogenous Retroviral Elements. Brain Behav. Immun..

[154] Balestrieri E., Pitzianti M., Matteucci C., D’Agati E., Sorrentino R., Baratta A., Caterina R., Zenobi R., Curatolo P., Garaci E. (2014). Human Endogenous Retroviruses and ADHD. World J. Biol. Psychiatry.

[155] Chiara C., Bernanda P.M., Claudia M., Elisa D., Tony M.M., Valentina R., Sandro G., Paolo C., Paola S.-V., Augusto P. (2018). The Decrease in Human Endogenous Retrovirus-H Activity Runs in Parallel with Improvement in ADHD Symptoms in Patients Undergoing Methylphenidate Therapy. Int. J. Mol. Sci..

[156] Gruchot J., Herrero F., Weber-Stadlbauer U., Meyer U., Küry P. (2023). Interplay between Activation of Endogenous Retroviruses and Inflammation as Common Pathogenic Mechanism in Neurological and Psychiatric Disorders. Brain Behav. Immun..

[157] Perron H., Lazarini F., Ruprecht K., Péchoux-Longin C., Seilhean D., Sazdovitch V., Créange A., Battail-Poirot N., Sibai G., Santoro L. (2005). Human Endogenous Retrovirus (HERV)-W ENV and GAG Proteins: Physiological Expression in Human Brain and Pathophysiological Modulation in Multiple Sclerosis Lesions. J. Neurovirol..

[158] Vargas D.L., Nascimbene C., Krishnan C., Zimmerman A.W., Pardo C.A. (2005). Neuroglial Activation and Neuroinflammation in the Brain of Patients with Autism. Ann. Neurol..

[159] Bergallo M., Galliano I., Montanari P., Zaniol E., Graziano E., Calvi C., Alliaudi C., Daprà V., Savino F. (2020). Modulation of Human Endogenous Retroviruses –H, -W and -K Transcription by Microbes. Microbes Infect..

[160] Römer C. (2021). Viruses and Endogenous Retroviruses as Roots for Neuroinflammation and Neurodegenerative Diseases. Front. Neurosci..

[161] Canli T. (2019). A Model of Human Endogenous Retrovirus (HERV) Activation in Mental Health and Illness. Med. Hypotheses.

[162] Curtin F., Perron H., Kromminga A., Porchet H., Lang A.B. (2015). Preclinical and Early Clinical Development of GNbAC1, a Humanized IgG4 Monoclonal Antibody Targeting Endogenous Retroviral MSRV-Env Protein. MAbs.

[163] Levet S., Medina J., Joanou J., Demolder A., Queruel N., Réant K., Normand M., Seffals M., Dimier J., Germi R. (2017). An Ancestral Retroviral Protein Identified as a Therapeutic Target in Type-1 Diabetes. JCI Insight.

[164] Curtin F., Vidal V., Bernard C., Kromminga A., Lang A.B., Porchet H. (2016). Serum Pharmacokinetics and Cerebrospinal Fluid Concentration Analysis of the New IgG4 Monoclonal Antibody GNbAC1 to Treat Multiple Sclerosis: A Phase 1 Study. MAbs.

[165] Irfan S.A., Murtaza M., Ahmed A., Altaf H., Ali A.A., Shabbir N., Baig M.M.A. (2022). Promising Role of Temelimab in Multiple Sclerosis Treatment. Mult. Scler. Relat. Disord..

[166] Curtin F., Bernard C., Levet S., Perron H., Porchet H., Médina J., Malpass S., Lloyd D., Simpson R. (2018). A New Therapeutic Approach for Type 1 Diabetes: Rationale for GNbAC1, an Anti-HERV-W-Env Monoclonal Antibody. Diabetes Obes. Metab..

[167] Curtin F., Champion B., Davoren P., Duke S., Ekinci E.I., Gilfillan C., Morbey C., Nathow T., O’Moore-Sullivan T., O’Neal D. (2020). A Safety and Pharmacodynamics Study of Temelimab, an Antipathogenic Human Endogenous Retrovirus Type W Envelope Monoclonal Antibody, in Patients with Type 1 Diabetes. Diabetes Obes. Metab..

[168] Morandi E., Tanasescu R., Tarlinton R.E., Constantin-Teodosiu D., Gran B. (2019). Do Antiretroviral Drugs Protect from Multiple Sclerosis by Inhibiting Expression of MS-Associated Retrovirus?. Front. Immunol..

[169] Gold J., Goldacre R., Maruszak H., Giovannoni G., Yeates D., Goldacre M. (2015). HIV and Lower Risk of Multiple Sclerosis: Beginning to Unravel a Mystery Using a Record-Linked Database Study. J. Neurol. Neurosurg. Psychiatry.

[170] McCormick A.L., Brown R.H., Cudkowicz M.E., Al-Chalabi A., Garson J.A. (2008). Quantification of Reverse Transcriptase in ALS and Elimination of a Novel Retroviral Candidate. Neurology.

[171] Gold J., Rowe D.B., Kiernan M.C., Vucic S., Mathers S., van Eijk R.P.A., Nath A., Garcia Montojo M., Norato G., Santamaria U.A. (2019). Safety and Tolerability of Triumeq in Amyotrophic Lateral Sclerosis: The Lighthouse Trial. Amyotroph. Lateral Scler. Front. Degener..

[172] Cipriani C., Giudice M., Petrone V., Fanelli M., Minutolo A., Miele M.T., Toschi N., Maracchioni C., Siracusano M., Benvenuto A. (2022). Modulation of Human Endogenous Retroviruses and Cytokines Expression in Peripheral Blood Mononuclear Cells from Autistic Children and Their Parents. Retrovirology.

